# Van der Waals Heterostructures—Recent Progress in Electrode Materials for Clean Energy Applications

**DOI:** 10.3390/ma14133754

**Published:** 2021-07-05

**Authors:** Chance Blackstone, Anna Ignaszak

**Affiliations:** Department of Chemistry, University of New Brunswick, Fredericton, NB E3B 5A3, Canada; chance.blackstone@unb.ca

**Keywords:** van der Waals heterostructure, electrocatalyst, metal–air batteries, fuel cell, electrolyzers, oxygen reduction and evolution, hydrogen evolution

## Abstract

The unique layered morphology of van der Waals (vdW) heterostructures give rise to a blended set of electrochemical properties from the 2D sheet components. Herein an overview of their potential in energy storage systems in place of precious metals is conducted. The most recent progress on vdW electrocatalysis covering the last three years of research is evaluated, with an emphasis on their catalytic activity towards the oxygen reduction reaction (ORR), oxygen evolution reaction (OER), and hydrogen evolution reaction (HER). This analysis is conducted in pair with the most active Pt-based commercial catalyst currently utilized in energy systems that rely on the above-listed electrochemistry (metal–air battery, fuel cells, and water electrolyzers). Based on current progress in HER catalysis that employs vdW materials, several recommendations can be stated. First, stacking of the two types vdW materials, with one being graphene or its doped derivatives, results in significantly improved HER activity. The second important recommendation is to take advantage of an electronic coupling when stacking 2D materials with the metallic surface. This significantly reduces the face-to-face contact resistance and thus improves the electron transfer from the metallic surface to the vdW catalytic plane. A dual advantage can be achieved from combining the vdW heterostructure with metals containing an excess of d electrons (e.g., gold). Despite these recent and promising discoveries, more studies are needed to solve the complexity of the mechanism of HER reaction, in particular with respect to the electron coupling effects (metal/vdW combinations). In addition, more affordable synthetic pathways allowing for a well-controlled confined HER catalysis are emerging areas.

## 1. Introduction

Climate change caused by rising global carbon emissions has driven the need for greener sources of energy and high-performance energy storage systems [1,2,3,4,5]. Commercial metal–air batteries, metal-ion batteries, proton exchange membrane fuel cells (PEMFCs), and electrolyzers rely on precious metals, such as palladium, iridium, lithium, and platinum, to drive their internal processes [6,7,8]. Rising costs, limited worldwide reserves, and geopolitical instability in dominant mining regions all severely decrease the viability of precious metal reliant energy storage systems to fulfill large-scale power grid roles [9,10,11]. Hence, cheaper and more abundant materials are top priority, even at a slight cost to performance.

With a need for more affordable, easy-to-scale-up, and yet well-performing electrochemical materials, the rising questions are how van der Waal materials could potentially replace currently utilized battery components? What problems could they eventually solve once fully developed and integrated into clean energy technologies?

For fuel cells and electrolyzers, the main obstacle is the cost of platinum needed for top-notch performance. An additional obstacle is sluggish kinetics of oxygen reduction at the cathode and the chemical and electrochemical degradation of fuel cell components due to highly corrosive electrolytes coupled with high operating potentials. Each issue limits the long-cycle life of these energy sources, even if the best noble platinum is utilized. Therefore, the development of vdW-based materials that can eventually replace platinum-based electrodes is driven by the need for much cheaper, abundant, and easy-to-be synthesized catalysts.

In the last ten years, van der Waals heterostructures have demonstrated unprecedented electrocatalytic activity. In contrast to currently published reviews on vdW materials [12,13,14,15,16], this study surveys the progress made in the last three years (2019–2021) and compares 2D-tailored vdW configurations and their intriguing electrocatalytic activities as electrodes in clean energy applications. Our aim is to carefully examine the aggregated advancements in vdW heterostructures, specifically utilized in the area of metal–air battery, and PEM fuel cell, and electrolyzers, and provides an overview as well as a critical insight into their applicability. Understanding the catalytic mechanism taking place with vdW-based electrocatalysts helps to uncover their potential to be utilized as affordable, highly active, and yet electrochemically stable electrode materials that could potentially compete with the most active Pt-based electrodes.

## 2. Van der Waals Heterostructures

**Figure 1:**
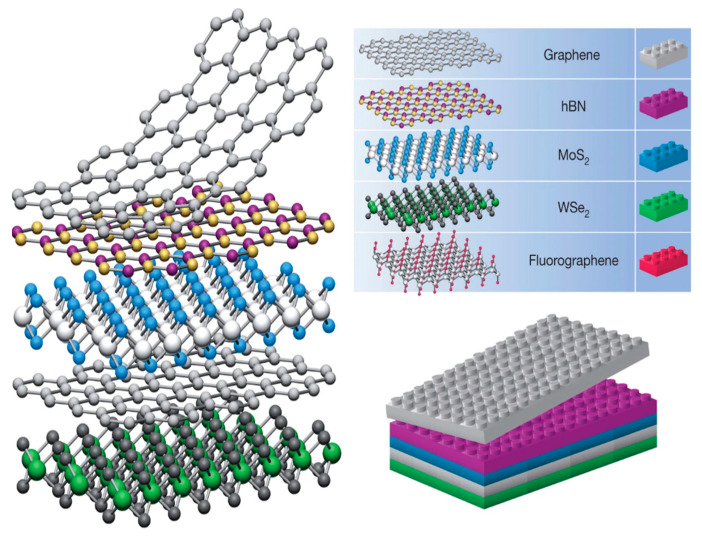
Structure of van der Waals heterostructures, similar to that of Legos. Reprinted (adapted) from reference [17], copyright (2013) Springer Nature.

2D sheets are less than 100 nm in a single coordinate direction. This specifies them as a thin paper or sheet-like material with a relatively expansive basal plane. Common examples are graphene (Gr), molybdenum disulfide (MoS_2_), and hexagonal boron nitride (hBN) [17]. Their unique structures induce anomalous chemical and electrochemical properties [18,19]. The synthetic methods of vdW materials have been recently reviewed by Di Bartolomeo [13], and therefore will not be detailed in this paper. In general, we can observe that despite the fact that many sophisticated methods have been developed for the synthesis of vdW materials, they can be fabricated in a rather easy way. One of the most frequently discussed is an exfoliation or “scotch tape” method categorized as a top-down approach. A 2D sheet may be exfoliated from its 3D crystal counterpart using this method. A bottom-up approach focuses on growing 2D sheets from small molecule precursors [18]. van der Waals heterostructures are stacks of 2D sheets illustrated in Figure 1. The basal planes of each sheet are held strongly together by covalent bonding, while van der Waals forces keep them fixed in a sort of sandwich structure. It is not an underestimation that vdW structures can be projected as atomic-scale Lego blocks. As perfect as Lego pieces match each other, vdW building blocks fit exceptionally well. Electronic coupling at the interface unlocks unique molecular characteristics, which are presumably tunable depending on the 2D sheet materials used, and other physical properties, such as layering and inter-layer distance [16]. Stability and contaminant issues heighten the difficulty of synthesizing heterostructures and limit the amount of available 2D building blocks. Typically, high stability of the 3D bulk crystal is required before one can begin peeling off 2D sheets [17].

### 2.1. Dimensional Materials

**Figure 2:**
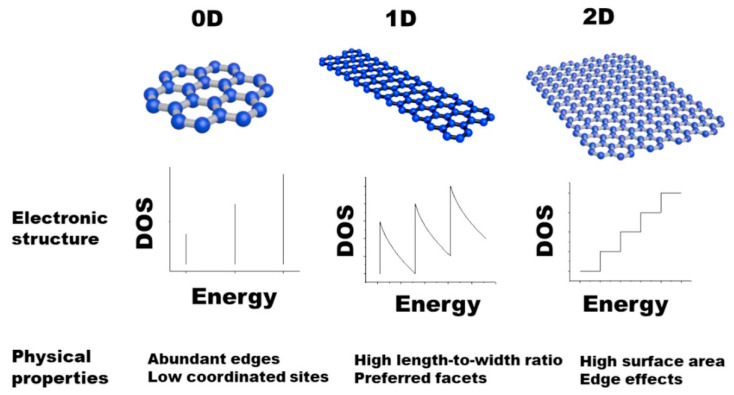
Density of states (DOS) of 0D, 1D, and 2D materials plotted vs. energy and correlated with physical properties of corresponding structures. Reprinted (adapted) from reference [18], copyright (2020) Elsevier.

When materials are smaller than 100 nm in at least one direction, induced changes in the electronic structure and surface properties give rise to unique molecules with different physical and chemical traits than their bulk material counterpart. The number of dimensions in which the molecule is confined results in its classification as 0D, 1D, or 2D [18], shown in Figure 2. If we consider the pathways of electron transport in these structures, the flow of electrons is then confined in zero, one, and two dimensions. This brings a significantly different distribution of density of states (DOS) versus energy, as demonstrated in Figure 2 (such as a staircase curve for 2D materials, a fence for 1D material, and a pack of discrete lines for 0D materials). For example, in atomic aggregates, clusters, or quantum dots that are categorized as 0D materials, the electrons travel in three directions (x, y, z) while in 1D materials (nanotubes, nanorods, nanoribbons, and nanowires) and 2D structures (thin nanofilms and nanosheets, graphene, graphene oxide), the movement of the electron is restricted to two and one directions, respectively. The nature of low-dimensional materials means they subsume many exciting catalytic characteristics, such as vast amounts of surface area active sites leading to high atom utilization, low coordinated environment, and unique electronic characteristics [18,20].

**Figure 3:**
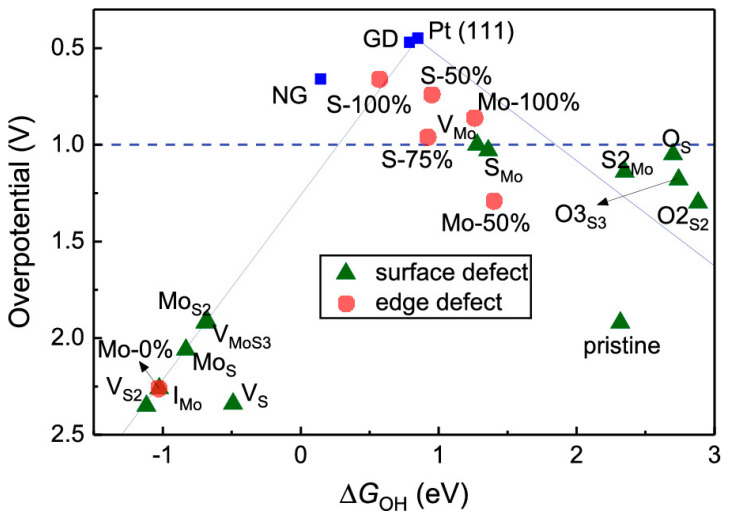
Relationship between overpotential of ORR process and ΔG_OH_ (ΔG_OH_ is adsorption free energies for the OH adsorbates intermediating the ORR processes) for defective MoS_2_. The data for graphene defect (GD), Pt(111), and N-doped graphene (NG) are provided for comparison. Reprinted (adapted) with permission from [23]. Copyright (2019) American Chemical Society.

For example, 2D materials have risen as promising catalysts for oxygen reduction reaction (ORR). In total, graphene (Gr), transition metal dichalcogenides (TMDs), such as MoS_2_, MoSe_2_, transitional metal oxides (TMOs), hexagonal boron nitride (h-BN), and metal organic frameworks (MOFs), have all shown potential to be utilized as efficient and affordable catalysts in energy systems that operate on oxygen electroactivity (i.e., fuel cells, metal–air battery, water electrolyzers). One excellent study that correlates dimensionality with catalytic activity is a work performed by Sihrostami et al. In particular, absorption energies of key ORR and oxygen evolution reaction (OER) intermediate over a variety of 2D materials was analyzed using density functional theory [21]. One of the most important findings revealed in this work is that several 2D structures have shown similar OH and OOH binding energies to Pt, one of the best ORR catalysts, and thus they become emerging materials that can potentially replace very expensive noble metal electrodes in clean energy systems. These materials include the N-doped version of Stone–Wales defects in graphene [22], MoS_2_ [23], or h-BN/Cu [24]. Figure 3 summarizes the relationship between overpotential of ORR process and adsorption energy of OH intermediates (ΔG_OH_) for defective MoS_2_ [23]. The data for graphene defect (GD), Pt(111), and N-doped graphene (NG) are provided for comparison. The second important conclusion from this work [21] is that binding energies largely follow the relationships observed for pure metals. Therefore, scaling relationships for these 2D materials indicate that the same models used for evaluating transition metal catalysts are also applicable for this class of materials. These established correlations can significantly accelerate the screening of van der Waals materials studied to date.

### 2.2. Confined Catalysis

**Figure 4:**
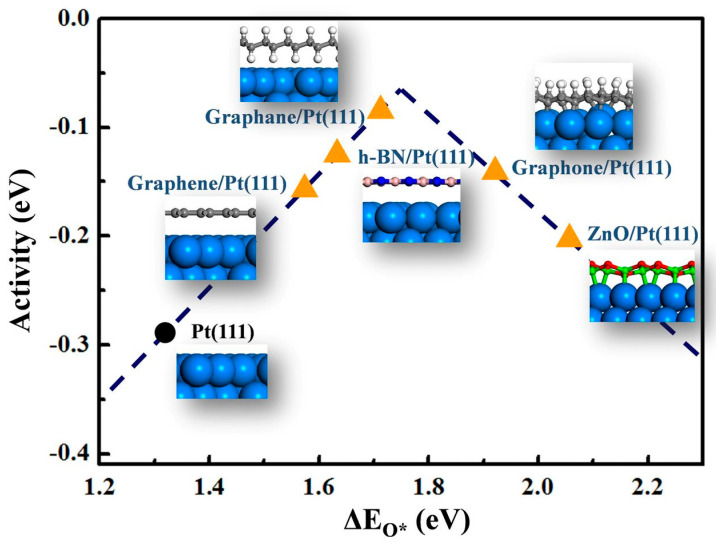
The volcano curve relation between ORR activity and binding energies of O atoms (ΔEO*) on Pt surface. Insets demonstrate the interfacial structures. B, purple balls; C, gray balls; H, white balls; N, dark blue balls; O, red balls; Pt, light blue balls; and Zn, green balls. Reprinted (adapted) with permission from [26]. Copyright (2017) National Academy of Sciences.

Confined catalysis takes place in a spatially constrained environment. Confined molecules experience a loss of entropy and interfacial effects due to the nanoconfinement effect, which can significantly alter their properties [25]. This was demonstrated practically by Haobo et al., who studied the mechanisms of pure platinum catalysis versus 2D graphene-coated platinum [26]. Absorption energies of CO were calculated for both molecules, differences between the two being attributed to the energy of confinement. Coated graphene was demonstrated to have weakened CO absorption. Furthermore, it was shown that potential energy increases slightly with the interlayer distance. This works to destabilize absorbed molecules. The effects were replicated with different platinum planes, suggesting that they are due to van der Waals interactions amongst the layers. Finally, ORR catalytic ability was shown to be tunable based on the properties of the 2D overlayer. The binding of oxygen in the interlayer was weaker than on pure platinum—notable because the ability of platinum as an ORR catalyst can be limited by how strongly it absorbs oxygen. The extent to which dissociative absorption energy can be reduced was dependent on the 2D overlayer species. This study concluded that 2D confined surface formed between 2D material overlayers and underlying metal surfaces can be used to explore confined catalysis, and that it can strongly influence geometric constraints for adsorbed molecules (Figure 4). In summary, the 2D overlayer makes the metal surface less energetically stable for adsorbed gas species. The geometric constraint and confinement field generated in the 2D space result in weaker adsorption on the metal surface. Clearly, this study demonstrated that surface reactivity and ORR activity can be altered on Pt coated with various 2D planes. This concept can be exploited in designing new, more active nanocatalysts composed of 2D materials and metals [26]. For the correlation shown in Figure 4, the binding energy is used as a descriptor of ORR activity. The theoretical (calculated) activity of the oxygen reduction reaction (ORR) expressed in energy barrier units (eV) is plotted as a function of the oxygen-binding energy. The y-axis is the activity and originates from −ΔG of the different reaction steps in the ORR process. For each step, the reaction free energy ΔG is defined as the difference between free energies of adsorption of the initial and final states of corresponding intermediate species: *O, *OOH, and *OH. Further details with respect to the methodology applied in the estimation of theoretical activity for ORR are discussed by Sun et al. [27].

## 3. Electrochemical Oxygen Reduction Reaction (ORR) and Evolution (OER): vdW Cathode Materials for a Metal–Air Battery and Fuel Cell

Metal–air batteries are cheap, can be utilized as primary and rechargeable systems, and have high capacities [6,28,29]. The main components are a metal anode, basic electrolyte, charge separator, and a gas diffusion cathode [6]. The metal anode, commonly made from either zinc, aluminum, magnesium, lithium, or iron [6,29], undergoes an electron releasing oxidation reaction. The resulting metal oxides subsequently diffuse within the basic electrolyte while the electrons go onward to power the connected system. Structurally the cathode consists of a carbon covering combined with a precious metal catalyst, such as platinum, which facilitates the oxygen reduction reaction (ORR; Equation (1)) as electrons generated at the anode, converge with oxygen absorbed from the environment [6]. Over time depletion of the solid metal anode, or a build-up of complexing solid metal hydroxide, hinder anodic reactions, which drives the need for battery replacement or refurbishing [6].

Proton exchange membrane fuel cells (PEMFCs) require a steady supply of fuel to produce current, while metal–air batteries are semi-sealed cells that contain all the necessary materials to drive their internal reactions. Hydrogen gas is oxidized at the anode, spurring the flow of electrons through the connected system. Protons flow from anode to cathode via the proton exchange membrane, a jelly-like electrolyte that is an excellent H^+^ conductor. Oxygen is reduced at the cathode generating water, and because this is the only product of their operation, PEMFCs are viewed as incredibly green sources of electricity [30,31,32]. Applying electricity to a PEMFC drives the operation in a backward manner, H_2_O now undergoes the oxygen evolution reaction, and H_2_ is produced at the cathode. Units designed solely for this purpose are called proton exchange membrane water electrolyzers. The concurrent use of both systems demonstrates an elegant method of renewable energy storage. Green hydrogen is the term used to describe H_2_ produced using renewables solely. Stored hydrogen gas may then be consumed in a PEMFC to power the grid or a home [31,32]. Other applications of PEMFCs have been electric vehicles (FCEV, fuel cell electric vehicles), large transportation systems (ships, tracks), and stationary power backup units. Their distinct advantage over metal-ion electric vehicles is a reduction in downtime. Metal-ion EVs must be recharged, while FCEVs need only to be refueled [9]. The major obstacles currently faced by hydrogen technology are the lack of H_2_ storage and refueling infrastructure and that the multistep process of H_2_ production, H_2_ storage, and energy production presents ample opportunity for efficiency losses [33].

The oxygen reduction reaction (ORR; Equation (1)) and oxygen evolution reaction (OER; Equation (2)) are pertinent chemical processes in the above-mentioned energy storage systems and the overall water splitting process. The slow reaction kinetics of ORR engender its dependence on precious metal catalysts, which in turn diminishes the suitability of the energy storage devices discussed in this work for large-scale applications [18,34]. Bifunctional electrocatalysts are needed to facilitate both ORR and OER in the development of lightweight, rechargeable metal–air batteries and dual-function fuel cells [35,36,37,38,39,40].

ORR     2H_2_O + 4e^−^ + O_2_ → 4OH (1)

OER     2H_2_O → 4H^+^ + 4e^−^ + O_2_ or 4OH^−^ → 2H_2_O + 4e^−^ + O_2_ (2)

The thorough analysis of van del Waals-structured ORR and OER electrocatalysts covering the most important discoveries until 2018 has been demonstrated by Yu et al. [12]. In total, research was conducted on validation of various vdW materials, including doped-graphene and its derivatives, transition metal dichalcogenides, transition metal-based structures, 0D/2D, 1D/2D, and 2D/2D hybrids, showed many intriguing characteristics. For example, self-supported 1D/2D heterostructures (i.e., electrocatalyst doped with nitrogen and oxygen through the layer-by-layer assembly of graphene and CNTs) demonstrated highly efficient transport and favorable catalytic kinetics, as well as excellent durability with little performance loss. Another important conclusion drawn from this work is that the stacking of two 2D/2D structures results in the modulation of contact resistance and the charge transport on the catalytic performance of van der Waal materials [12], and research discussed in this review highlighted that catalytic activity of 2D materials can be further improved through the construction of 2D/2D architectures, taking advantage of the activity of its individual components. The most recent outlook of ORR/OER vdW catalysts developed until 2019 was discussed by Yan et al. [16], concluding that for both ORR and OER improved electrocatalytic performance is attributable to the large active surface area and unique electronic coupling between the combination of vdW heterostructures, such as N-doped MoS_2_ and graphene. With respect to advancements in OER catalyst, most of the recent work evolves around photocatalytic OER utilizing MoS_2_/ZnO or P/BSe vdW structures and improving the charge recombination at the heterojunctions [16], rather than evaluating OER catalysis taking place during the charging of the metal–air battery. Thus, this section covers exclusively only the state-of-the-art vdW cathode catalyst for ORR/OER reported within 2018–2021.

**Figure 5:**
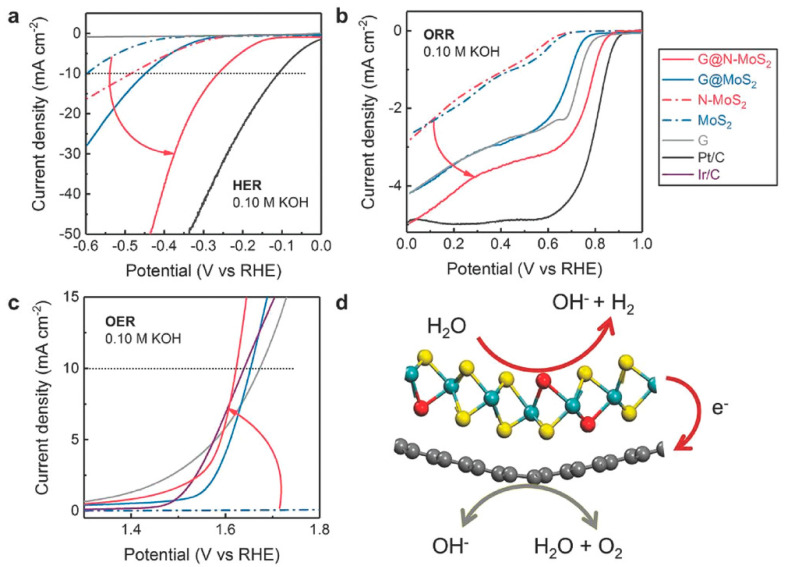
(**a**) HER polarization curves obtained in N_2_-saturated 0.10 M KOH; (**b**) ORR, and (**c**) OER polarization curves in O_2_-saturated 0.10 M KOH solution; (**d**) the electron transfer effects in G@N-MoS_2_ heterostructures for ORR, OER, and HER. Reprinted (adapted) from [41] with permission from John Wiley and sons.

Mesoporous, nitrogen-doped MoS_2_/Gr heterostructures synthesized by Tang et al. displayed promising hydrogen evolution (HER; Equation (3)) activity, as well as promising ORR and OER activity, potentially making them a trifunctional catalyst [41]. Trials carried out in 0.10 M KOH demonstrated a limiting current density that approached the Pt/C catalyst and a reduced half-wave potential difference for ORR. In OER, the activity was shown to be competitive with the Ir/C Catalyst, Figure 5. Compared to conventional reference catalysts, such as Pt/C and Ir/C, the Gr/N-doped MoS_2_ exhibited unique trifunctional activity in 0.10 M KOH. Besides excellent HER activity, the ORR limiting current density for this catalyst approaches that of Pt/C (Figure 5b). Moreover, the OER activity is within the range of the precious Ir/C catalyst (Figure 5c). Notably, the ORR activities for pure Gr/MoS_2_ (no nitrogen doping) and Graphene plus MoS_2_ were found to be lower than that of pure graphene. This indicates that nitrogen-doped MoS_2_ may exhibit inferior ORR and OER activity to the mesoporous graphene. Therefore, the heightened activity of the Gr/N-MoS_2_ was attributed to strengthening effects from the coupling interactions. 

**Figure 6:**
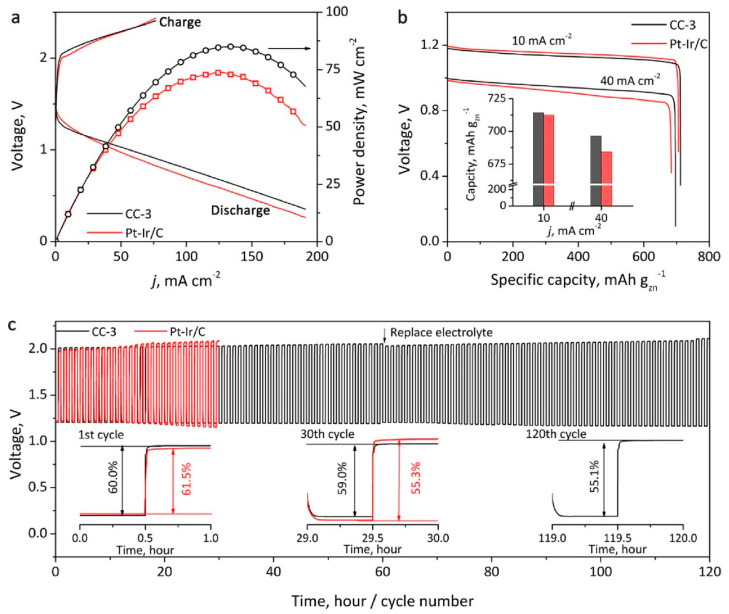
(**a**) Galvanodynamic charge/discharge profiles and power density and (**b**) galvanostatic discharge curve for rechargeable zinc–air batteries assembled using CC-3 and Pt–Ir/C oxygen catalysts. The inset shows the battery capacity under different discharge current densities. (**c**) Cycling profiles. The insets show cycling performances at 1st, 30th, and 120th cycles. Reprinted from [42] with permission from American Chemical Society Copyright © 2021.

A 1D van der Waals heterostructure composed of a carbon nanotube core, coated with thienothiophene-pyrene was recently synthesized by Liu et al. [42]. In proposed CNT/COF structures, the COF shell was controlled by tuning the mass ratio between CNTs and COF precursors (m_CNT_/m_precursors_ = 1, 2, or 4). The resulting 1D vdW structures are denoted as CC-X, where X refers to the thickness of the COF shell estimated by TEM imaging. The thickness of the COF shell was manipulated, and the results suggested a significant impact on electrochemical performance in response. Theoretical analyses revealed that the carbon–sulfur region within the thienothiophene group is the active site of catalysis. A COF thickness of 3 nm was determined to be optimal for catalytic performance, giving a half-wave potential of 0.828 V for ORR and a Tafel slope of 101 mV dec^−1^ for OER. The electrochemical characteristics of a metal–air battery constructed of CNT/COF-based ORR catalysts were compared to the same system made of a conventional Pt-Ir/C catalyst (Figure 6 [42]). For example, the battery prototype with a CC-3 catalyst delivered a power density that was 21% higher than that of the Pt−Ir/C. Under the same discharging current, the capacity of the CC-3-based battery was 714 mAh g_Zn_^−1^ that was comparable to 712 mAh g_Zn_^−1^ of the Pt−Ir/C (Figure 6b). Under a high discharging current density of 40 mA cm^−2^, the CC-3-based battery outperformed the Pt−Ir/C system (696 vs. 684 mAh g_Zn_^−1^, Figure 6b). The zinc–air battery cycling performance was evaluated by 120 discharging/charging cycles, as demonstrated in Figure 6c. Both battery prototypes showed similar characteristics at the first cycle (inset on the left in Figure 6c). After 30 cycles, the efficiency of the CC-3 system declined slightly to 59.0%, while that of the Pt−Ir/C-based cell quickly dropped to 55.3%. After 120 cycles, the CC-3 battery still retained a high efficiency of 55.1% [42].

### Prospective of van der Waals Heterostructures as ORR/OER Catalyst for Metal–Air and Fuel Cell Cathodes

Despite the significant progress made on vdW electrocatalysts for the ORR and OER, there are still some challenges to overcome before vdW are transferred to fuel cell and metal–air battery commercialization. Several important areas for future research into the exploration of van der Waal catalysts are proposed below:

(1) Establishing a relationship between the electrode catalyst support and the vdWs is important to realize the effective utilization of vdWs from their distribution on the support. Possible approaches include incorporating the support in the formation of the vdWs during the synthesis or combining the support with the as-synthesized heterostructures. It is well-known that optimizing the catalyst loading and the catalyst/support interaction can further improve the electrochemical performance.
(2) Both bottom-up and top-down synthesis procedures must be more scalable and more energy efficient in order to make the commercialization of these catalysts viable and benefit the corresponding energy systems.
(3) Structural stability under the ORR/OER conditions is an emerging area of research that must be completed. Particularly, the chemical instability of vdWs in alkaline solution, surface segregation, Kirkendall effect, vdW aggregation between layers, and its connection to supporting material must be ensured to maintain a contact activity during long-life electrochemical processes.
(4) Lastly, the vdW-based catalyst layer of the commercial electrodes, such as air cathode or membrane electrode assembly (MEA), should be developed and optimized. In particular, more research is needed to ensure the best choice for the catalyst support material resulting in the highest utilization of vdWs, and thus sufficient/or improved performance when compared to commercialized fuel cells and metal–air batteries.

## 4. Electrochemical Hydrogen Evolution (HER): vdW Electrodes for Water Splitting

The hydrogen evolution reaction (HER) is an integral part of the overall water splitting process. As covered in this chapter, H_2_ gas production can act as a method of storing intermittent renewable energy sources. Proton exchange membrane water electrolyzers facilitate water splitting, with the HER reaction occurring at the cathode, producing the desired hydrogen gas. The unique physical and electrochemical properties of vdW heterostructures make them an intriguing option for potential HER catalysts. In reality, their anisotropic nature makes a pure sample a poor catalytic choice [43,44,45,46]. Atoms are firmly bonded in a satisfied coordination environment leading to an inert structure. Only edge atoms have the necessary properties to act as HER catalytic sites. Therefore, vdW heterostructures rely heavily upon defects, such as atomic vacancies, heteroatom doping, and lattice deformation, to greatly enhance their ability as HER catalysts. Currently, water splitting systems are highly dependent upon precious metals, such as platinum [47].

HER     2H^+^ + 2e^−^ → H_2_ (3)

The emerging vdW heterostructures, considering their application in photocatalysis, photovoltaics, phot-electrochemical water splitting, have been summarized by Di Bartolomeo et al. [13], Yu et al. [12], and Hu et al. [14]. The most important conclusions on HER catalysts based on vdW structures discovered until now are that: (i) graphene like- or its derivatives, doped-graphene quantum dots (GQDs), are the most researched vdW heterostructures, (ii) and that new materials, such as α-CSe/β-CSe, CdS/g-C_3_N_4_ or g-ZnO/WS_2_, are predicted to show promise in HER catalysis based on theoretical prognosis. In the preceding section, we offered an update of the most recent HER for electrochemical water splitting with emphasis on electrochemical characteristics, excluding the review on vdWs photocatalyst that is broadly covered by other authors.

**Figure 7:**
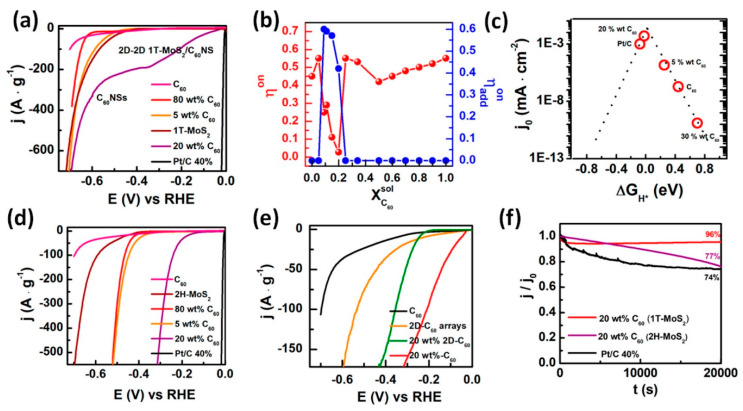
(**a**) Mass-standardized current density for C_60_, 1T-MoS_2_, commercial Pt/C 40%, and the 1T-MoS_2_/C_60_ heterostructures with 5, 20, and 80 wt.% of C_60_ in 0.5 M H_2_SO_4_. (**b**) HER onset overpotential values as a function of the C_60_ volume fraction in solution. (**c**) Volcano plots of the as-synthesized vdW 1T-MoS_2_/C_60_. (**d**) Mass-standardized catalytic currents for C_60_, 2H-MoS_2_, commercial Pt/C 40%, and the 2H-MoS_2_/C_60_ heterostructures with 5, 20, and 80 wt.% of in 0.5 M H_2_SO_4_. (**e**) Mass-standardized LSVs for C_60_, C_60_ NSs, 20 wt.% 2D-C_60_, and 20 wt.% C_60_ in 0.5 M H_2_SO_4_. (**f**) Chronoamperometric curves of 2H-MoS_2_/20 wt.% C_60_ and 1T-MoS_2_/20 wt.% C_60_ at −0.35 V vs. RHE. Reprinted from [49], Copyright (2020) with permission from American Chemical Society.

Molybdenum disulfide, graphene heterostructures (MoS_2_/Gr), have shown particular promise as HER catalysts [40,48,49]. MoS_2_ is a highly abundant metal and a fair catalyst by itself, exhibiting thermoneutral hydrogen absorption free energy and stability in acidic solutions [48]. It usually exists as a 2D layered material, in which sheets of covalently bound S-Mo-S are stacked on top of one another. Active sites for hydrogen absorption are abundant on the sulfur-dominated edges of the material [50]. The catalytic ability of pure MoS_2_ is limited by two major drawbacks, both of which conduce poor atom utilization. The first being poor conversion efficiency since the catalyst is saturated when approximately only 25% of active sites are covered in H_2_ [48]. Second, the vast majority of atoms are contained within an inert basal plane that contains no active sites to contribute towards catalysis [50]. van der Waals heterostructures with graphene enhance catalytic performance compared to pure MoS_2_ by increasing the interlayer spacing, thereby exposing more active sites and improving electrical conductivity [40,48,49,51]. Yu et al. was able to synthesize a MoS_2_/Gr heterostructure via a three-step process and tracked its performance in 0.5 M H_2_SO_4_ and 0.5 M KOH [48]. Although the heterostructure was unable to outperform the standard Pt/C HER catalyst, it did show significant improvement over MoS_2_, boasting overpotentials of 180 mV and 263 mV at electric current densities of 10 mA cm^−2^ and 100 mA cm^−2^, plus a Tafel slope of 79 mV dec^−1^ in the acidic solution and in basic solution, an overpotential of 183 mV at an electric current density of 10 mA cm^−2^ and Tafel slope of 127 mV dec^−1^. All tests were performed vs. a reversible hydrogen electrode. Nitrogen-doped MoS_2_, graphene mesoporous heterostructures (G@N/MoS_2_) were examined by Tang et al. [40]. A two step-sequential CVD-based process was employed to synthesize the product. Tafel slopes were collected to analyze HER kinetics, which came out to 82.5 mV dec^−1^. The active sites on this heterostructure are modified by the strong interaction between mesoporous graphene substrate and N-MoS_2_; this brings about favorable absorption energies and improved HER kinetics. Finally, MoS_2_/C_60_ van der Waals heterostructures were synthesized via a one-pot process and identified using Raman and X-ray photoelectron spectroscopy by Santiago et al. [49]. The electrode characteristics are compared to the commercial Pt/C catalyst and demonstrated in Figure 7. For example, the onset potentials of the vdW-based electrodes were plotted as a function of the C_60_ weight content (Figure 7b). Among tested vdW materials, 1T-MoS_2_/20 wt.% C_60_ displayed a Tafel slope that was comparable to the conventional Pt/C catalyst. This result was accompanied by the position of vdW catalyst in the volcano plots shown in Figure 7c. 1T-MoS_2_/20 wt.% C_60_ catalyst was found in the proximity of the Pt/C peak, revealing similar catalytic activity of both. In addition, other MoS_2_/C_60_ combinations also demonstrated HER activity that was comparable to that of commercial system. This was correlated with the structure of C_60_ and the presence of well-ordered fullerene nanosheets that are either forming free-MoS_2_ domains or intercalated into the MoS_2_ layers. The onset overpotentials of the 2D-2D 1T-MoS_2_/C_60_ NS domains shown in Figure 7e also demonstrate very efficient HER electrocatalysis. The better catalysis on these vdW catalyst was related to the improved conductivity of the 2D-2D 1T-MoS_2_/C_60_ NS framework that overall facilitates electron transfer to the catalytically active sites, and to its optimal interfacial interactions that decrease the energy barriers of each catalytic step, thus improving the HER activity. The 1T-MoS_2_/20 wt.% C_60_ structures also showed great stability that suppressed that of conventional Pt/C in the same experimental conditions (Figure 7f, [49]).

**Figure 8:**
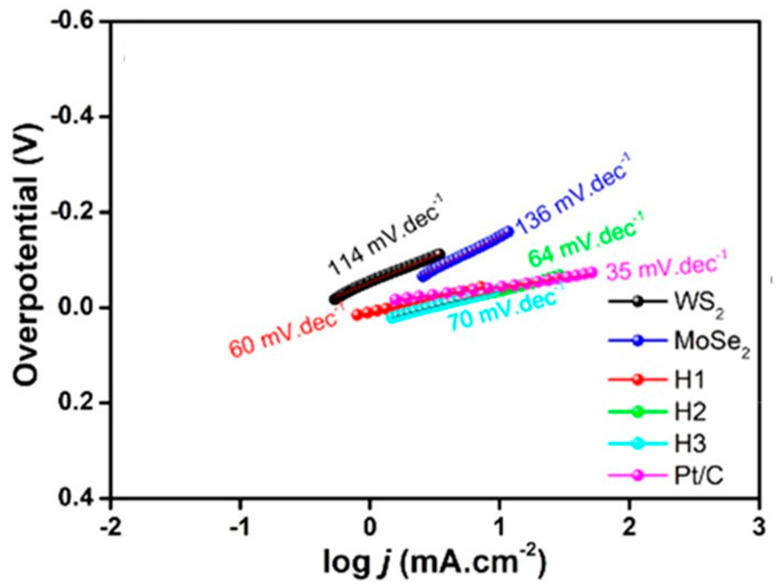
Tafel plots for each material tested by Vikraman et al. Reprinted from reference [7], with permission from ACS Publications.

Non-graphene-based van der Waals heterostructures consisting of molybdenum and tungsten dichalcogenides layers have also been examined as possible HER catalysts [7,51]. The rate-determining step is the absorption of hydrogen onto active sites of the catalyst [52], which is directly tied to the Gibbs free energy associated with the process. A computational study of MoS_2_/WS_2_ revealed two potential active sites dubbed S1 and S2, correlating to a sulfur site with elongated bonds and a sulfur site with compressed bonds. Within the interlayer, the Gibbs free energy of each active site was noticeably reduced to 0.20 eV for S1 and 0.24 eV for S2, showcasing the structure’s catalytic potential [51]. Very recently, a MoSe_2_/WS_2_ heterostructure was synthesized by Vikraman et al. using a dual preparation method of chemical bath and chemical vapor deposition (CVD) [7]. MoSe_2_ sheets were deposited on ultrasonically etched conducting fluorine-doped tin oxide (FTO) within a chemical bath. Tungsten was coated to the MoSe_2_-layered FTO via a radio frequency sputtering system. The films were then sulfurized using CVD techniques at 500 °C. Three different heterostructures were made, each run increasing the sputtering time, subsequently increasing the thickness of the top layer WS_2_. The as-prepared heterostructures made up the working electrode of a three-electrode system used to evaluate HER performance. Ag/AgCl acted as a reference electrode, and a graphite rod acted as a counter electrode, while 0.5 M H_2_SO_4_ was used as an electrolyte. The Tafel plots for each tested material shown in Figure 8 are fitted from the raw data. The Tafel slopes of the heterostructures H1 (30 min sputtering), H2 (20 min sputtering), and H3 (40 min sputtering) are vastly improved compared to the sheets of MoSe_2_ and WS_2_ on their own. It should be noted that the traditional Pt/C electrode was still the best performing catalyst with a Tafel slope value of 35 mV dec^−1^. Thus, less overpotential is required to increase the kinetics of the HER reaction.

### Prospective of van der Waals Heterostructures as the HER Electrocatalysts

The above discussed new vdW heterostructures offer a great foundation to develop water splitting systems to generate H_2_ fuel. Based on current progress in this area, several recommendations can be stated. First, stacking of the two types vdW materials (this accounts for pairing 0D/2D, 1D/2D, and 2D/2D) with one being graphene or its doped derivatives results in significantly improved HER catalysis. This can be attributed to more efficient charge transport facilitated by a more conductive component. More importantly, the subtle contact resistance between different vdW structures can be accomplished for 2D materials that were synthesized separately via wet chemistry methods. This major breakthrough inspires research for using a template-assisted synthesis procedure, allowing the control of both the macroscopic features of HER electrodes (e.g., porosity and gas permeability) together with a molecular-level design (e.g., formation of a high number of active catalytic sites in 2D space). The second important recommendation is to take advantage of an electronic coupling when stacking 2D materials with the metallic surface. First, this significantly reduces the face-to-face contact resistance and thus improves the electron transfer from the metallic surface to the vdW catalytic plane. A dual advantage can be achieved by combining the vdW heterostructure with metals containing an excess of d electrons (e.g., gold). As a result, electrons injected from the metal to a basal plane of vdW catalyst can accelerate both the charge transport and, at the same time, improve the adsorption of hydrogen on the basal plane of vdW catalysts. Despite these recent and promising discoveries, more studies are needed to solve the complexity of the mechanism of HER reaction, in particular with respect to the electron coupling effects (metal/vdW combinations). Furthermore, more affordable synthetic pathways allowing for a well-controlled confined HER catalysis are emerging areas. These challenges should inspire research groups to create more combinations of vdW heterostructures with other 0D, 1D, and 2D counterparts while embracing the best catalytic features of each component.

## 5. Conclusions

van der Waals heterostructures have shown their capability as possible electrode materials and electrocatalysts. van der Waals heterostructures have been shown to catalyze HER, ORR, and OER much better than their 2D counterparts owing to their unique electrochemical properties, and in some cases, rivaling precious metal catalysts, such as platinum and iridium [7,33,41,42,48,49,50]. Graphene and MoS_2_ based van der Waals heterostructures have shown particular promise as a trifunctional catalyst of all three reactions [41]. 2D sheets of transition metal dichalcogenides studied, such as MoS_2_, MoSe_2_, and WS_2_, are relatively stable on their own, which helps ease the synthesis of their heterostructures [17].

Moving forward, studies on van der Waals heterostructures should focus on expanding the current library of materials, engineering more sophisticated cells with heterostructure electrodes, and improving synthetic processes for highly controllable layer and heteroatom doping to achieve fine-tuning of catalytic activity.

## Data Availability

Not applicable.
